# Antagonist of Growth Hormone-Releasing Hormone Potentiates the Antitumor Effect of Pemetrexed and Cisplatin in Pleural Mesothelioma

**DOI:** 10.3390/ijms231911248

**Published:** 2022-09-24

**Authors:** Iacopo Gesmundo, Francesca Pedrolli, Nicoletta Vitale, Alessia Bertoldo, Giulia Orlando, Dana Banfi, Giuseppina Granato, Ramesh Kasarla, Federico Balzola, Silvia Deaglio, Renzhi Cai, Wei Sha, Mauro Papotti, Ezio Ghigo, Andrew V. Schally, Riccarda Granata

**Affiliations:** 1 Division of Endocrinology, Diabetes and Metabolism, Department of Medical Sciences, University of Turin, 10126 Turin, Italy; 2 Molecular Biotechnology Center, Department of Molecular Biotechnology and Health Sciences, University of Turin, 10126 Turin, Italy; 3 Department of Oncology, University of Turin, 10126 Turin, Italy; 4 Division of Gastroenterology, Azienda Ospedaliero-Universitaria Città della Salute e della Scienza di Torino, University of Turin, 10126 Turin, Italy; 5 Department of Medical Sciences, University of Turin, 10126 Turin, Italy; 6 Immunogenetics and Transplant Biology Service, Città Della Salute e Della Scienza University Hospital, 10126 Turin, Italy; 7 Endocrine, Polypeptide, and Cancer Institute, Veterans Affairs Medical Center, Miami, FL 33125, USA; 8 South Florida VA Foundation for Research and Education, Miami, FL 33125, USA; 9 Pathology Unit, Città Della Salute e Della Scienza University Hospital, 10126 Turin, Italy; 10 Divisions of Hematology/Oncology and Endocrinology, Department of Medicine, University of Miami Miller School of Medicine, Miami, FL 33101, USA; 11 Sylvester Comprehensive Cancer Center, University of Miami Miller School of Medicine, Miami, FL 33136, USA

**Keywords:** GHRH antagonists, pleural mesothelioma, chemotherapeutic drugs

## Abstract

Pleural mesothelioma (PM) is an aggressive cancer with poor prognosis and no effective therapies, mainly caused by exposure to asbestos. Antagonists of growth hormone-releasing hormone (GHRH) display strong antitumor effects in many experimental cancers, including lung cancer and mesothelioma. Here, we aimed to determine whether GHRH antagonist MIA-690 potentiates the antitumor effect of cisplatin and pemetrexed in PM. In vitro, MIA-690, in combination with cisplatin and pemetrexed, synergistically reduced cell viability, restrained cell proliferation and enhanced apoptosis, compared with drugs alone. In vivo, the same combination resulted in a strong growth inhibition of MSTO-211H xenografts, decreased tumor cell proliferation and increased apoptosis. Mechanistically, MIA-690, particularly with chemotherapeutic drugs, inhibited proliferative and oncogenic pathways, such as MAPK ERK1/2 and cMyc, and downregulated cyclin D1 and B1 mRNAs. Inflammatory pathways such as NF-kB and STAT3 were also reduced, as well as oxidative, angiogenic and tumorigenic markers (iNOS, COX-2, MMP2, MMP9 and HMGB1) and growth factors (VEGF and IGF-1). Overall, these findings strongly suggest that GHRH antagonists of MIA class, such as MIA-690, could increase the efficacy of standard therapy in PM.

## 1. Introduction

Pleural mesothelioma (PM) is a rare and aggressive cancer, generally diagnosed at an advanced stage and characterized by a poor prognosis, with median overall survival between 6 and 12 months [1]. PM develops in the thin layer surrounding the mesothelium and is mainly caused by the inhalation of asbestos fibers, with a long latency of 20–50 years between exposure to asbestos and the onset of the disease. Inhaled asbestos fibers cause inflammation of the pleura, DNA damage of mesothelial cells with recruitment of macrophages and neutrophils, leading to activation of proliferative and oncogenic pathways and tumorigenesis [2]. Histologically, PM has been classified into three major subtypes: epithelioid (50–70% of cases), sarcomatoid (10–20% of cases) and biphasic, a combination of epithelioid and sarcomatoid (30% of cases) [3]. Of note, sarcomatoid mesothelioma has a poor prognosis, with a median survival of 4 months, while epithelioid and biphasic PM have better outcomes, of 13.1 and 8.4 months, respectively [1,4]. The first-line systemic treatment for unresectable PM is the combination of cisplatin and pemetrexed [2,5,6]. However, PM is often refractory to standard chemotherapy, with response rates of only 40% and a median overall survival of 12 months, likely because of tumor aggressiveness, resistance of mesothelioma cells to chemotherapy and immune-suppressive environment [1,7]. Another novel strategy is represented by the combination of cisplatin and pemetrexed with the antiangiogenic agent bevacizumab [8], which, however, has not received approvals from US FDA or European Medicines Agency. Moreover, novel biomedical devices for use with platinum-pemetrexed and immunotherapy agents have been recently approved for the treatment of PM, especially in patients with non-epithelioid tumors, who have the greater magnitude of survival benefit [6,9]. However, the therapeutic options for PM are not resolutive to permit the complete recovery of affected patients; therefore, new treatment strategies are urgently needed.

Growth hormone-releasing hormone (GHRH) is a hypothalamic neuropeptide that regulates the release of growth hormone (GH) from the pituitary gland [10]. In addition, GHRH exerts direct stimulatory effects in extrapituitary cells and tissues through binding to GHRH receptors (GHRH-Rs). It has been also demonstrated that GHRH and its agonistic analogs display cardioprotective, anti-inflammatory, anxiolytic and antidiabetogenic effects, and promote proliferation, migration and survival in many non-malignant and tumor cell types. Moreover, the expression of GHRH and GHRH-Rs, including the splice variant 1 (SV1) of GHRH-R, has been shown in a variety of cancers, where GHRH acts as a growth factor through paracrine/autocrine-mediated mechanisms [11,12,13,14,15,16,17,18]. Thus, the GHRH/GHRH-R pathway represents a suitable target for the treatment of cancer [19]. Consequently, many antagonists of human GHRH have been synthesized and tested by other groups [20], and by the laboratory of one of us (A.V.S.) in recent decades [13], showing their capacity to suppress the growth of experimental cancers in vitro and in vivo, through the blockade of the stimulatory loop produced by tumor-derived GHRH and GHRH-Rs. More recently, the antagonists of Miami (MIA) class and AVR class were developed, endowed with increased binding affinity for GHRH-Rs, compared with previous classes of antagonists, and only weak endocrine GH inhibitory activity [21,22]. Among these analogs, MIA-602 and MIA-690 showed potent suppressive effects on the growth of many cancers, including lung, gastric and colorectal cancers, where they exerted anti-inflammatory, antioxidative and proapoptotic functions [23,24,25]. Importantly, MIA-602, also reduced inflammation and fibrosis in a mouse model of lung injury, by inhibiting inflammatory pathways, such as signal transducer and activator of transcription 3 (STAT3) and nuclear factor kappa-B (NF-kB) [26,27], and decreased inflammation in sarcoidosis-like granuloma [28]. Furthermore, we recently demonstrated that MIA-602 and MIA-690, as single agents, inhibit the growth of human PM cell lines and primary cells in vitro, and exert strong antitumor effects in vivo in PM mice, by blunting proliferative and oncogenic pathways, as well as expression of tumor insulin-like growth factor 1 (IGF-1) and vascular endothelial growth factor (VEGF) [29]. However, the antitumor role of GHRH antagonists in PM in vivo in combination with chemotherapeutic compounds, remains to be investigated. Thus, based on these premises and considering the need for more effective therapies for PM, we aimed to verify whether GHRH antagonist MIA-690 potentiates the antitumor effect of standard therapy, i.e., cisplatin and pemetrexed, in mice xenografted with biphasic PM cell lines. Furthermore, we examined the mechanisms involved in the effects of the antagonists, either alone or in combination.

## 2. Results

### 2.1. MIA-690 Increases the Inhibitory Effects of Cisplatin/Pemetrexed on Cell Viability of PM Cells

**Figure 1:**
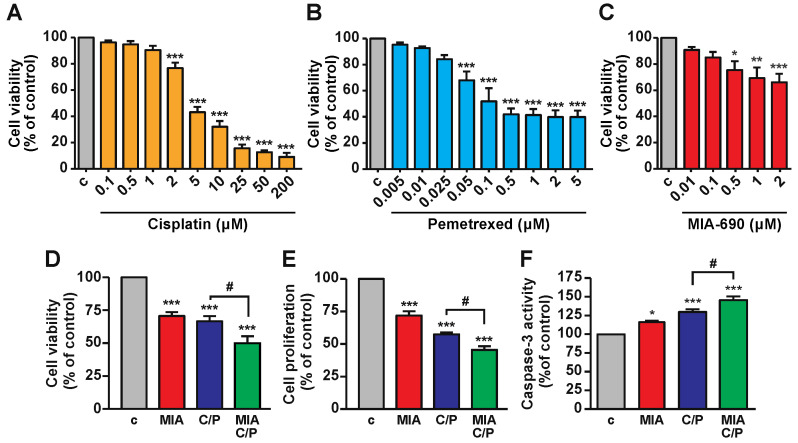
Inhibitory effects of cisplatin, pemetrexed and MIA-690, alone or in combination, in PM cells. Cell viability, assessed by MTT in MSTO-211H cells cultured for 48 h in DMEM containing 2.5% serum in either absence (c, control) or presence of cisplatin (**A**), pemetrexed (**B**) or MIA-690 (**C**) at the concentration indicated. Results, expressed as percent of control, are means ± SEM. * *p* < 0.05, ** *p* < 0.01 and *** *p* < 0.001 vs. c by one-way ANOVA and Dunnet’s multiple comparison post-hoc test (*n* = 5); each condition was performed in quintuplicate. Cell viability (**D**), cell proliferation (**E**) and apoptosis (**F**) assessed by MTT, BrdU and caspase-3 activity assays, respectively, in MSTO-211H cells cultured in DMEM + 2.5% serum either without (c, control) or with 1 μM MIA-690 (MIA), 2 μM cisplatin/50 nM pemetrexed (C/P) or the three compounds in combination (MIA/C/P), at the same concentrations. Results, expressed as percent of control, are means ± SEM. * *p* < 0.05, *** *p* < 0.001 vs. c; ^#^ *p* < 0.05 by one-way ANOVA and Tukey’s multiple comparison post-hoc test (*n* = 6 for **D** and *n* = 4 for **E** and **F**); each condition was performed in quintuplicate.

To select the best inhibitory concentrations of the different compounds, dose-response experiments were initially performed at 48 h in MSTO-211H human biphasic cell lines, representative of the epithelioid/sarcomatoid PM histological subtype, already employed in our previous study [29]. Cisplatin was tested at concentrations ranging from 0.1 to 200 μM (Figure 1A), in line with former analysis performed in these cells [30], showing a dose-dependent inhibition of cell viability. Pemetrexed, tested at 0.005 to 5 μM, was effective from 0.05 to 5 μM (Figure 1B), while MIA-690, analyzed at 0.01 to 2 μM, significantly decreased cell viability from 0.5 to 2 μM (Figure 1C), as previously demonstrated [29].

Based on cell viability experiments, where different concentrations were tested in combination (data not shown), 1 μM MIA-690, 2 μM cisplatin and 50 nM pemetrexed were selected as best concentrations for the subsequent experiments. Treatment for 48 h with MIA-690 reduced both cell viability and proliferation, and increased apoptosis, an effect observed also with cisplatin/pemetrexed alone. The addition of MIA-690 to cisplatin/pemetrexed produced a further inhibition of both cell viability and proliferation, as well as an elevation in apoptosis, compared with cisplatin/pemetrexed alone (Figure 1D–F). The combination index (CI) value of MIA-690/cisplatin/pemetrexed for cell viability was 0.33, indicating strong synergistic effect versus either MIA-690, cisplatin or pemetrexed alone. These findings demonstrate that MIA-690 potentiates the antitumor effect of chemotherapy in PM cell lines.

### 2.2. MIA-690 Potentiates the In Vivo Antitumor Effect of Cisplatin/Pemetrexed in PM Xenografts

**Figure 2:**
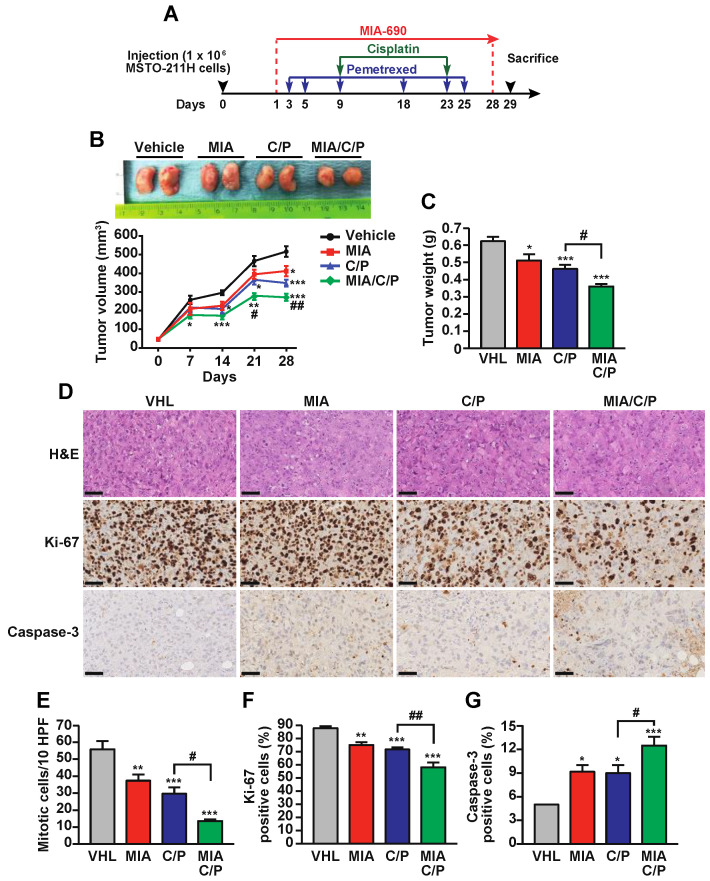
Inhibitory effects of MIA-690 in PM in vivo in combination with cisplatin and pemetrexed. (**A**) Experimental protocol: at day 1, when MSTO-211H tumor volume was over 40 mm^3^, mice were treated for 28 days with either s.c. injection of 5 μg/day MIA-690, i.p. injection with cisplatin and pemetrexed (C/P) (5 mg/kg and 100 mg/kg, respectively) at the days indicated, or the combination of MIA/C/P, at the same concentrations. Mice were sacrificed at day 29. (**B**) Representative PM tumors isolated at day 29 from mice treated as described above. The graph shows tumor growth curves. Results are means ± SEM. * *p* < 0.05, ** *p* < 0.01, *** *p* < 0.001 vs. vehicle; ^#^ *p* < 0.05, ^##^ *p* < 0.01 vs. C/P by two-way ANOVA and Tukey’s multiple comparison post-hoc test (*n* = 9 in each group). (**C**) Tumor weight in PM mice. Results are means ± SEM. * *p* < 0.05, *** *p* < 0.001 vs. vehicle (VHL); ^#^ *p*< 0.05 (*n* = 9 in each group) by one-way ANOVA and Tukey’s multiple comparison post-hoc test. (**D**) Representative sections of MSTO-211H tumors from each group of animals, stained with hematoxylin and eosin (H&E), Ki67 and cleaved caspase-3. Scale bars, 100 µm. (**E**) Mitotic cell number counted in 10 high power fields (HPF) in H&E-stained sections. (**F**) Percentage of Ki67 positive cells. (**G**) Percentage of caspase-3 positive cells. Results for E-G are means ± SEM. * *p* < 0.05, ** *p* < 0.01, *** *p* < 0.001 vs. VHL; ^#^ *p* < 0.05, ^##^ *p* < 0.01 by one-way ANOVA and Tukey’s multiple comparison post-hoc test. (*n* = 6 in each group).

The efficacy of GHRH antagonist MIA-690 in combination with cisplatin/pemetrexed was subsequently evaluated in vivo, in non-obese diabetic (NOD)/ severe combined immune deficient (SCID)/gamma chain^−/−^ mice xenografted with MSTO-211H cells, that received 4-week treatment with the different compounds (Figure 2A). Nine animals were studied in each of the following groups: (1) vehicle, (2) MIA-690, (3) cisplatin/pemetrexed, (4) MIA-690/cisplatin/pemetrexed. In line with our previous findings [29], daily subcutaneous (s.c.) injection of 5 μg MIA-690 inhibited the growth of PM by reducing tumor volume at days 21 and 28 by 15% and 20%, respectively, compared with vehicle. Furthermore, coadministration of 5 mg/kg cisplatin and 100 mg/kg pemetrexed, injected intraperitoneally (i.p.), blunted tumor growth by 22% at day 21 and by 26% at day 28. Interestingly, MIA-690 in combination with cisplatin/pemetrexed produced a stronger inhibitory effect by 35% and 47% versus vehicle, and by 15% and 18% versus cisplatin/pemetrexed alone, at day 21 and 28, respectively (Figure 2B).

Similarly, tumor weight was decreased at the end of the experiment after treatment with MIA-690 as single agent or cisplatin/pemetrexed, by 18% and 26%, respectively, and was further reduced by the combination of MIA-690 with the drugs (58% reduction versus vehicle and 22% versus cisplatin/pemetrexed) (Figure 2C). No variation in body weight was observed in mice, at week 1 and 23 of treatment, in all experimental conditions (Supplementary Figure S1). Immunohistochemistry analysis revealed that MIA-690 and, to a greater extent, cisplatin/pemetrexed, reduced tumor cell proliferation, as assessed by decreased number of both mitotic and Ki-67 positive cells, while increasing apoptosis, as indicated by enhanced staining for cleaved caspase-3. These effects were potentiated by MIA-690 plus cisplatin/pemetrexed, compared with cisplatin/pemetrexed alone (Figure 2E–G). Overall, these results indicate that MIA-690, besides exerting antitumor effect *per se*, enhances the in vivo inhibitory effect of chemotherapy in PM. 

### 2.3. The Combination of MIA-690 with Cisplatin and Pemetrexed Is More Effective than Chemotherapeutic Drugs Alone on the Inhibition of Proliferative and Survival Pathways 

**Figure 3:**
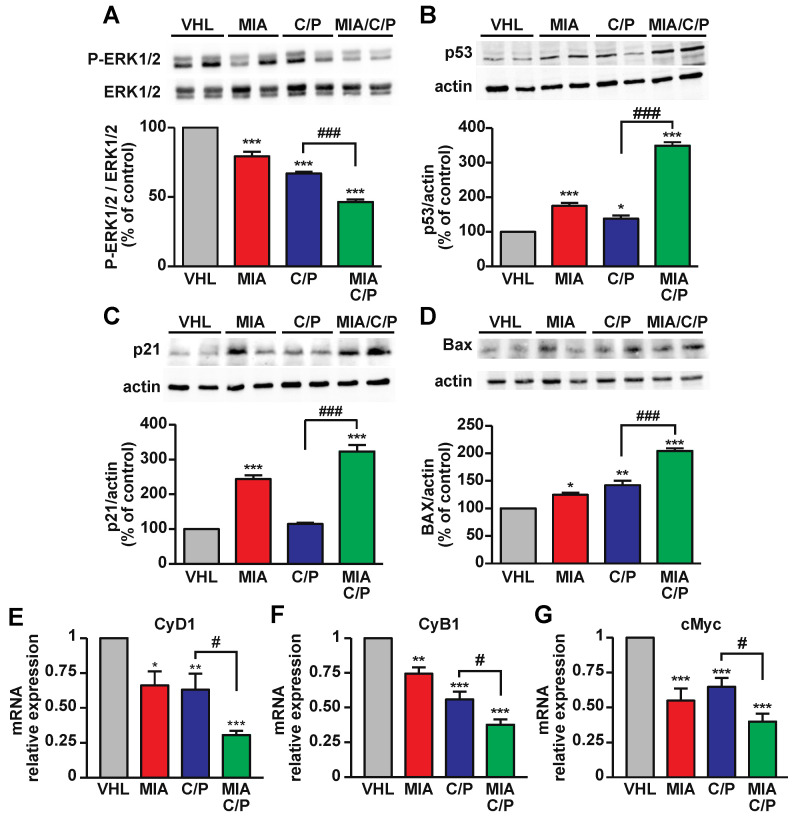
The role of MIA-690 in combination with chemotherapeutic drugs on proliferative and apoptotic pathways and cell cycle regulators in PM tumors. Representative Western blots of phosphorylated ERK1/2 (P-ERK1/2) (**A**), p53 (**B**), p21 (**C**) and Bax (**D**) in PM specimens (top) of mice treated with vehicle (VHL), MIA-690, cisplatin and pemetrexed (C/P) or the combination of MIA/C/P. Blots, were reprobed with ERK1/2 (**A**) or actin (**B**–**D**) for normalization (bottom). Results are expressed as percentage of control (VHL) (means ± SEM). * *p* < 0.05, ** *p* < 0.01, *** *p* < 0.001 vs. VHL; ^###^ *p* < 0.001 by one-way ANOVA and Tukey’s post hoc test (*n* = 4 for each group). Real-time PCR analysis for cyclin D1 (CyD1) (**E**), cyclin B1 (CyB1) (**F**) and cMyc (**G**) mRNA in tumor tissues, normalized to 18S rRNA (means ± SEM). * *p* < 0.05, ** *p* < 0.01, *** *p* < 0.001 vs. VHL; ^#^ *p* < 0.05 by one-way ANOVA and Tukey’s post hoc test (*n* = 6 for each group).

To examine the proliferative pathways involved in the antitumor effects of MIA-690 in combination with cisplatin/pemetrexed, Western blot and real-time PCR analysis were performed in tumor tissues. The activation of mitogen-activated protein kinases (MAPK) extracellular signal-regulated kinase 1/2 (ERK1/2), a critical regulator of cell proliferation and survival in many tumors, including PM [31], was reduced by treatment with MIA-690 alone, as well as by the combination of cisplatin/pemetrexed, compared with vehicle. Moreover, the addition of MIA-690 strongly reduced ERK1/2 phosphorylation versus both vehicle and drugs alone (Figure 3A). Consistently, MIA-690/cisplatin/pemetrexed potently increased the levels of the tumor suppressor p53 protein (Figure 3B) and its downstream target, the cyclin-dependent kinase inhibitor p21 (Figure 3C), both involved in cell cycle arrest and apoptosis [32]. Interestingly, MIA-690 as single agent upregulated p53 and p21, while chemotherapeutic drugs had little or no effect, compared with vehicle. In line with these findings and the increase in caspase-3 staining observed in tumor tissues, all treatments elevated the levels of Bax apoptotic protein, particularly in mice treated with MIA-690/cisplatin/pemetrexed, compared with chemotherapeutic agents alone (Figure 3D).

Many cancers, including PM, are closely related to cell cycle regulators such as cyclins, which are downregulated by p53/p21 and are promising targets for treatment-refractory cancers [33,34]. Real-time PCR results showed that the mRNA levels of cyclin D1 and cyclin B1, involved in the progression through G1 to S phases and G2/M transition, respectively, were reduced by either MIA-690 or cisplatin/pemetrexed. Moreover, both cyclins were potently inhibited by the three compounds in combination, compared with vehicle or drugs alone (Figure 3E,F). Similar effects were observed for cMyc oncogene, whose transcript levels were reduced by all treatments and, particularly, by MIA-690 plus cisplatin/pemetrexed (Figure 3G). Overall, these findings indicate that MIA-690, both *per se* and in combination with chemotherapy drugs, reduces PM tumor growth in vivo via the inhibition of proliferative/oncogenic pathways and activation of apoptosis. 

### 2.4. MIA-690 Increases the Anti-inflammatory Action of Cisplatin and Pemetrexed in PM Tumors 

**Figure 4:**
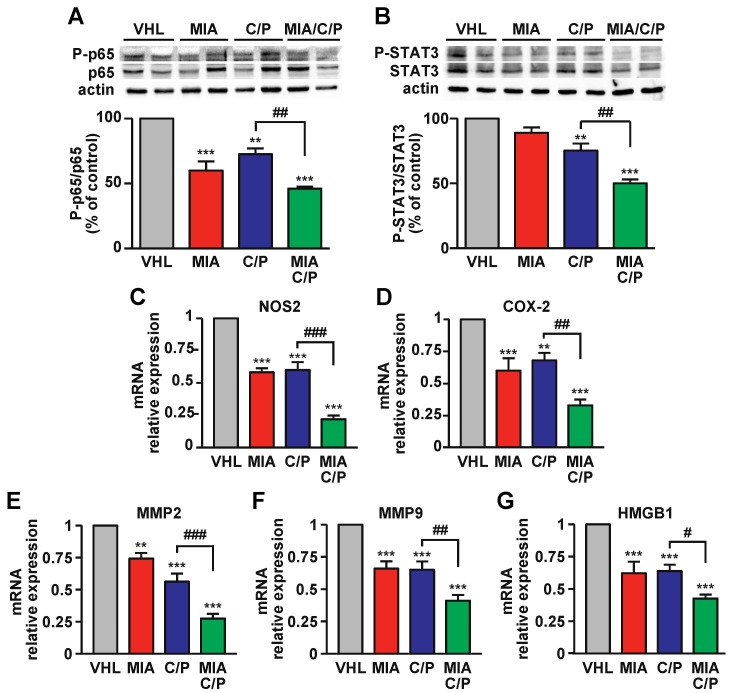
Anti-inflammatory action of MIA-690 in combination with chemotherapeutic drugs in PM tissues. Representative Western blots for phosphorylated NF-kB (P-p65) (**A**) and STAT3 (P-STAT3) (**B**) (top) in MSTO-211H xenografts of mice treated with vehicle (VHL), MIA-690, cisplatin and pemetrexed (C/P) or the combination of MIA/C/P. Blots were reprobed with respective total antibodies for normalization and for actin (bottom). Results, expressed as percentage of control (VHL), are means ± SEM. ** *p* < 0.01, *** *p* < 0.001 vs. VHL; ^##^ *p* < 0.01 by one-way ANOVA and Tukey’s post hoc test (*n* = 4 for each group). Real-time PCR analysis for iNOS (**C**), COX-2 (**D**), MMP2 (**E**), MMP9 (**F**) and HMGB1 (**G**) mRNA in PM tumors, normalized to 18S rRNA (means ± SEM). ** *p* < 0.01, *** *p* < 0.001 vs. VHL; ^#^ *p* < 0.05, ^##^ *p* < 0.01, ^###^ *p* < 0.001 by one-way ANOVA and Tukey’s post hoc test (*n* = 7 for each group).

The transcription factors NF-kB and STAT3 play a crucial role in inflammation, tumorigenesis, immunosuppression and chemoresistance and are highly expressed in many cancers, including PM [35,36]. Western blot analysis of PM tumors showed that treatment with MIA-690, either as single agent or with cisplatin/pemetrexed, strongly reduced the phosphorylation of the NF-kB p65 subunit, compared with both vehicle and drugs alone (Figure 4A). In line with this finding, STAT3 phosphorylation was inhibited by MIA-690/cisplatin/pemetrexed versus both vehicle and cisplatin/pemetrexed, while MIA-690 alone induced a slight but not significant inhibition (Figure 4B). Interestingly, MIA-690 combined with chemotherapy was more effective than drugs alone on inhibition of genes involved in inflammation and tumorigenesis, such as inducible nitric oxide synthase (iNOS) (Figure 4C), cyclooxygenase-2 (COX-2) (Figure 4D), metalloproteinase 2 (MMP2) (Figure 4E) and MMP9 (Figure 4F) and high mobility group box 1 (HMGB1) (Figure 4G). In addition, MIA-690 alone displayed the same effect as cisplatin/pemetrexed on inhibition of these genes, compared with vehicle. These results suggest that MIA-690 exerts anti-inflammatory actions in PM and potentiates those induced by chemotherapeutic drugs.

### 2.5. MIA-690 Potentiates the Effect of Cisplatin/Pemetrexed on Inhibition of VEGF and IGF-1 Expression in PM Tumors

**Figure 5:**
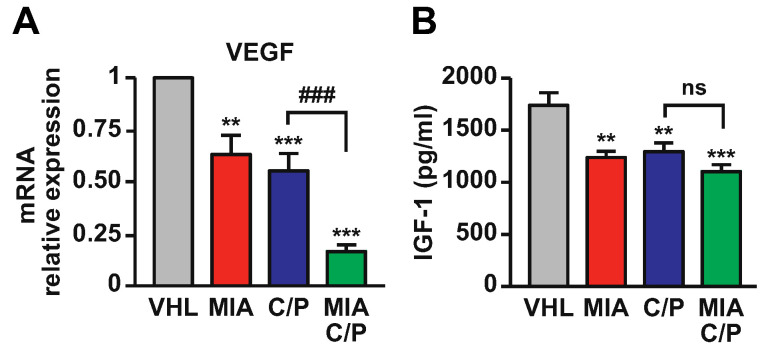
Inhibitory effect of MIA-690 in combination with cisplatin and pemetrexed on expression of VEGF and IGF-1 in PM xenografts. (**A**) VEGF mRNA levels assessed by real-time PCR and normalized to 18S rRNA. Results are means ± SEM. ** *p* < 0.01, *** *p* < 0.001 vs. VHL; ^###^ *p* < 0.001 by one-way ANOVA and Tukey’s post hoc test (*n* = 7 for each group). (**B**) Tumor IGF-1 protein levels measured by ELISA. Results are means ± SEM. ** *p* < 0.01, *** *p* < 0.001 vs. VHL; ns, not significant (*n* = 8 for each group).

We next investigated whether the combination of MIA-690 with cisplatin and pemetrexed would further induce the decrease in VEGF and IGF-1 expression in PM xenografts. As expected, MIA-690 as single agent reduced the levels of both VEGF mRNA and IGF-1 protein in tumors, compared with vehicle. A similar effect was observed in mice treated with cisplatin/pemetrexed. Surprisingly, VEGF mRNA levels were potently inhibited by MIA-690/cisplatin/pemetrexed compared with chemotherapeutic agents alone (Figure 5A). IGF-1 expression was slightly, but not significantly reduced by the combination of MIA-690 with the drugs, compared with drugs alone (Figure 5B). These results indicate that the combination of MIA-690 with chemotherapy produces antitumor effects through the inhibition of angiogenic pathways.

## 3. Discussion

Pleural mesothelioma is a rare cancer with a very poor prognosis, for which curative treatment modalities are still lacking. Here, we demonstrate that the GHRH antagonist MIA-690 potentiates the antitumor effects of the chemotherapeutic drugs cisplatin and pemetrexed, in vitro, in mesothelioma cells, and particularly in vivo, in mice PM xenografts. Of note, the in vivo effects of MIA-690 in combination with cisplatin and pemetrexed included the reduction in tumor volume and weight, inhibition of mitotic markers and increase in proapoptotic molecules. Furthermore, the activation of survival, proliferative and inflammatory pathways, the expression of oncogenic markers, cell cycle regulators and angiogenic factors, were blunted in PM tumors of mice treated with MIA-690/cisplatin/pemetrexed, compared with chemotherapy alone.

The standard treatment for unresectable PM consists of the combination of cisplatin and pemetrexed, which, however, has not proved to be resolutive and only partially improves the patients’ quality of life [2,6]; consequently, novel therapeutic strategies are needed. We have previously demonstrated that GHRH antagonists MIA-602 and MIA-690 alone exert potent and similar antitumor effects in vitro, in human PM cell lines and primary cells, and in vivo, in a xenograft model of human PM [29]. Thus, in light of these findings, we hypothesized that GHRH antagonists of MIA series would enhance the anticancer effect of chemotherapy in vivo. We initially confirmed the ability of MIA-690, as a single agent, to inhibit cell viability and proliferation and promote apoptosis in vitro in biphasic PM cells. Moreover, we first showed that the combination of MIA-690 with cisplatin/pemetrexed synergistically increased the antitumor effects of chemotherapeutic drugs alone, indicating that MIA antagonists sensitize PM cells to drug-induced toxicity. In addition, differently from our previous findings where the antitumor effect in vivo was assessed with the antagonists alone, we show here the ability of MIA-690 to enhance the inhibitory action of cisplatin/pemetrexed in mice PM xenografts. In keeping with these results, GHRH antagonists of JMR and MZ classes in combination with cytotoxic agents, including cisplatin and taxans, have been reported to inhibit the in vitro and in vivo growth of different experimental cancers, such as breast, lung and colon cancers [37,38,39,40].

The MAPK ERK1/2 pathway is involved in different cellular processes, e.g., survival and apoptosis, proliferation, differentiation and inflammation, and is often activated in many cancers, including mesothelioma, where it plays a key role in asbestos-induced carcinogenesis [31,41]. Previous studies demonstrated that GHRH antagonist JMR-132 inhibits the growth of human prostate cancer xenografted into nude mice through the inactivation of ERK [42]. In addition, it was revealed that GHRH antagonists MZ-J-7-138 or JV-1-92, in combination with docetaxel, reduced the ERK upstream protein K-RAS and COX-2 in mice xenografted with non-small cell lung cancer cells (NSCLCs) [40]. In line with these findings, we show here that MIA-690 reduced ERK1/2 phosphorylation in PM xenografts and further enhanced the inhibitory effect of chemotherapy. 

The p53 tumor suppressor protein has a prominent role in blocking cancer development by inducing apoptosis and cell cycle arrest, and promoting DNA repair and cell senescence. p53 also acts as a transcription factor to activate apoptotic proteins, such as Bax, and inhibits cell cycle regulators such as cyclin B1 and B2 [32]. Importantly, TP53, encoding p53, is the most commonly mutated gene in human cancers, and the deletion of Pten and TP53 in mouse mesothelium results in the development of non-epithelioid mesothelioma [43]. We have previously demonstrated that MIA-602 and MIA-690, in addition to displaying antitumor activities, increase the expression of p53 in human PM cell lines [29], as well as in GH/prolactin-secreting pituitary adenoma cell lines [44] and colorectal cancer cells [23]. Furthermore, earlier GHRH antagonists counteracted the mitogenic effects of GHRH in lung cancer cell lines by increasing p53 and inhibiting MAPK activation, as well as the expression of inflammatory iNOS, COX-2 and NF-kB [45]. In fact, the anticancer properties of GHRH antagonists are enhanced by their anti-inflammatory effects, associated with upregulation of p53 [46]. Here, the novel finding is that MIA-690 not only increased p53 levels in PM tumors, but also enhanced the effect of chemotherapy on p53 elevation. Similar results were observed for the expression of the cyclin-dependent kinase inhibitor p21, which is strongly induced by p53 and mediates p53-induced G1 cell cycle arrest [32]. Interestingly, the silencing of p21 expression in human non-small cell lung carcinoma (NSCLCs) cells abolished the antiproliferative effect of GHRH antagonist JMR-132 [47]. Moreover, a different study showed that JMR-132 inhibited the proliferation of colon cancer cells through p21 mediated S-phase arrest and increase in apoptosis [48]. Of note, it has been shown that the inactivation of p53 and consequent loss of p21 results in an increase in Ki-67 in human colon carcinoma cells [49]. Consistent with these findings, along with the elevation in p53 and p21 levels, we observed a decrease in cell proliferation and an increase in apoptosis in PM mice treated with both MIA-690 alone and in combination with chemotherapy, as demonstrated by the reduction in Ki67 and elevation in cleaved caspase-3 and Bax expression, respectively. 

Aberrant cell proliferation is one of the key hallmarks of cancer and a consequence of dysregulated cell division [50]; thus, targeting regulatory proteins involved in cell division is a potential approach to induce cell cycle arrest. Cyclin D1 and CDK4/6 (cyclin-dependent kinases 4 and 6) proteins are responsible for progression through G1 to S phases, a step which is often deranged in many cancers, including mesothelioma. Moreover, different transcription factors involved in mitogenic signaling, such as cMyc, STAT3 and NF-kB, promote the transcription of cyclin D genes [33,34,51]. Here, MIA-690, both alone and in combination with cisplatin/pemetrexed, strongly down-regulated the mRNA levels of cyclin D1 and cyclin B1, which are involved in G2/M transition of the cell cycle, suggesting inhibitory effects on cell cycle regulators. Accordingly, MIA-602 and MIA-690 were previously found to inhibit the growth and progression of lung cancer by modulating the levels of cyclin D1, D2 and CDKs [24].

Dysregulation of cMyc expression has been demonstrated in many human cancers, including mesothelioma [52]. Moreover, the inhibition of cMyc was found to increase the sensitivity to cisplatin and p21-activated kinase (PAK) inhibitors and to reduce proliferation in MSTO-211H cell lines [53,54]. In fact, cMyc promotes tumorigenesis by inducing cell proliferation, stimulating migration and angiogenesis, among other mechanisms. In addition, cMyc induces the expression of cell-cycle checkpoint genes, including cyclins, and regulators of apoptosis. In preclinical models, cMyc inactivation resulted in sustained tumor regression; thus, therapies targeting cMyc are promising for reversing cancer growth [55]. We have previously demonstrated the ability of MIA-602 and MIA-690 to inhibit in vitro PM cell proliferation and viability and cMyc expression [29], a result confirmed here in vivo, where mice treated with MIA-690, either alone or with cisplatin/pemetrexed, showed reduced cMyc expression in PM tumors. Accordingly, the anticancer effects of MIA-690 and the downregulation of cMyc were recently shown by our group in vitro, in pituitary adenoma cell lines [44], and in a collaborative study by Recinella et al. in colorectal cancer in mice, where an increase in p53 protein levels and inhibtion of COX-2, iNOS, and NF-kB gene levels were also observed [23]. Accordingly, our present results show that MIA-690 potentiates the effect of cisplatin/pemetrexed in PM xenografts also on inhibition of NF-kB, assessed as phosphorylation of p65 subunit, and STAT3 phosphorylation. The activation of NF-kB is involved in inflammatory pathways and associated with exposure to asbestos fibers and tumor evolution in mesothelioma [2]. Furthermore, the transcription factor STAT3, which controls the expression of genes regulating survival, proliferation and self-renewal, is frequently activated in many cancers, including mesothelioma, and is associated with oncogenic characteristics and poor survival [2,35]. Importantly, NF-kB and STAT3 physically and functionally interact in chemotherapy resistant MPM cell lines [56]. Moreover, MIA-602 was found to inhibit the growth of gastric cancer in vitro and in vivo by blocking p21-activated kinase 1 (PAK1), STAT3 and NF-kB inflammatory pathways [25]. In addition, NF-kB/p65 and COX-2 were found reduced by GHRH antagonists, along with a decrease in prostate weight, in experimental benign prostate hyperplasia [57]. Interestingly, it has been shown that p53 suppresses inflammation through inhibition of NF-kB, while activation of NF-kB resulted in p53 suppression [58]. Our present results also demonstrate the ability of MIA-690 to inhibit oxidative and inflammatory markers in PM tumors, such as iNOS and COX-2, particularly in combination with cisplatin/pemetrexed. Elevated expression levels of iNOS and COX-2 in tumor cells is associated with cell proliferation, invasion, resistance to apoptosis and the pathogenesis of different cancers, including mesothelioma [59,60,61]. Moreover, the inhibitory role of GHRH antagonists on these inflammatory pathways has been demonstrated in different types of experimental cancers [23,57], as well as inflammatory diseases [62].

Metalloproteinases are strongly implicated in most of the dysregulated processes in cancer, such as tumor growth, metastasis and angiogenesis and are highly expressed in mesothelioma, particularly MMP2 and MMP9 [63]. In agreement with our previous in vitro findings in PM cells [29], we show here that the in vivo treatment with MIA-690 inhibited the mRNA expression of both MMP2 and MMP9 in PM tumors, an effect elevated by the combination of MIA-690 with cisplatin/pemetrexed, compared with chemotherapy alone. Similar results were observed for expression of the proinflammatory protein HMGB1, which is released by necrotic mesothelial cells in response to asbestos and is a critical regulator in the initiation of inflammation and carcinogenesis [64]. In fact, HMGB1 stimulates the secretion of TNF-α from macrophages and activates protumoral factors such as NF-κB, leading to chronic inflammation, autophagy and mesothelial cell transformation [65]; thus, targeting HMGB1 may be an additional potential therapy for mesothelioma.

We also observed a reduction in VEGF mRNA levels in tumors of mice treated with either MIA-690 or cisplatin/pemetrexed alone, in line with our previous findings in PM xenografts [29]. This effect was increased by the combination of MIA-690 with cisplatin/pemetrexed versus drugs alone, confirming the antiangiogenic role of GHRH antagonists of MIA series. This finding suggests that GHRH antagonists may represent promising therapeutic molecules for precision medicine, in combination with specific inhibitors such as the antiangiogenic agent bevacizumab, which has been shown to be effective in selected PM patients in combination with cisplatin and pemetrexed [8].

Interestingly, the GHRH antagonist MZ-5-516 in prostate cancer cells [66], as well as MIA-602, MIA-606 and MIA-690 in non-small cell lung cancer cells [67], reduced VEGF secretion and attenuated the mRNA expression and activity of MMP2 and MMP9. In line with the results on VEGF secretion, MIA-690 alone reduced IGF-1 protein levels in PM tumors, suggesting direct effects, as previously demonstrated in both mesothelioma and lung cancer cells [29,40]. Moreover, IGF-I levels were further decreased by the combination of MIA-690 with chemotherapy, although this effect was not statistically significant compared with chemotherapy alone. Importantly, the dysregulation of IGF-1 receptor (IGF-1R) signaling is implicated in the growth and tumorigenesis of many cancers, including mesothelioma [68]. 

Overall, this study provides compelling evidence demonstrating that MIA-690 enhances the antitumor effects of cisplatin/pemetrexed in PM, both in vitro and particularly in vivo, where it acts by inhibiting key oncogenic, proliferative, inflammatory and angiogenic pathways, while increasing proapoptotic molecules. Thus, these novel results suggest that GHRH antagonists could improve the anticancer effectiveness of standard therapy in pleural mesothelioma.

## 4. Materials and Methods

### 4.1. Reagents 

The GHRH-R antagonist MIA-690 [PhAc-Ada^0^ -Tyr^1^, D-Arg^2^, Cpa^6^, Ala^8^, Har^9^, Fpa^510^, His^11^, Orn^12^, Abu^15^, His^20^, Orn^21^, Nle^27^, D-Arg^28^, Har^29^] hGH-RH(1-29)NH_2_ was synthesized and purified in the laboratory of one of the authors (Andrew V. Schally) at the Veterans Affairs Medical Center, University of Miami, Miami, FL, USA, as described previously [29,44]. For in vitro experiments, MIA-690 was dissolved in 100% dimethyl sulfoxide (DMSO) (MilliporeSigma, Milan, Italy) and diluted with appropriate incubation medium. The concentration of DMSO never exceed 0.1% (vol/vol). Pemetrexed was from Selleckchem (Houston, TX, USA) and cisplatin was from Tocris Bioscience (Bristol, UK). Thiazolyl blue tetrazolium bromide (MTT), RPMI-1640 medium, fetal bovine serum (FBS), bovine serum albumin (BSA), penicillin, amphotericin B, L-glutamine, primers and cell culture reagents were from MilliporeSigma. P-STAT3(9131S), P-NF-κB (3033S), NF-κB (8242S), P-ERK1/2 (9101S), ERK1/2 (9102S), and BAX (5023S) antibodies were from Cell Signaling Technology (Danvers, MA, USA). STAT3 (sc-482), p53 (sc-1313), p21 (sc-56335), and actin (sc-376421) antibodies were from Santa Cruz Biotechnology (Dallas, TX, USA). RT-PCR and Real-Time PCR reagents were from Invitrogen (Thermo Fisher Scientific, Milano, Italy).

### 4.2. Cell Culture

The human biphasic MPM cell line MSTO-211H was purchased from American Type Culture Collection (ATCC) (Manassas, VA, USA). Cells were maintained at 37 °C in a 5% CO_2_ humidified atmosphere in RPMI-1640 with 10% FBS, 2 mM L-glutamine, penicillin (100 U/mL), streptomycin (100 μg/mL) and 250 ng/mL amphotericin B.

### 4.3. Cell Survival and Proliferation

Cells were seeded in 96-well plates at the concentration of 2 × 10^3^ cells/well. After 48 h, cells were serum-starved for 12 h and incubated with the different stimuli for further 48 h. Cell survival was assessed by MTT assay. Cells were incubated with 1 mg/mL of MTT for approximately 2 h, then the medium was removed, and formazan products solubilized with 100 μL DMSO (MilliporeSigma). Cell viability was assessed by spectrophotometry at 570 nm absorbance using the LT-4000 microplate reader (Euroclone, Milan, Italy). Cell proliferation was assessed using the 5-bromo-2-deoxyuridine (BrdU) incorporation ELISA kit (Roche Diagnostic, Milan, Italy). Briefly, the cells were incubated with BrdU labeling solution for 2 h at 37 °C. After the removal of the labeling solution, cells were fixed, denatured, and incubated for 90 min with anti-BrdU antibody conjugate, which was subsequently removed by rinsing three times. Finally, cells were incubated in substrate solution at room temperature and proliferation assessed by colorimetric analysis at 450 nm absorbance using the LT-4000 microplate reader (Euroclone, Milan, Italy). 

### 4.4. Caspase-3 Activity

Cells were seeded into a 6-well plate at a concentration of 3 × 10^4^ cells/well. Caspase-3 activity was assessed by Caspase-3 Colorimetric Assay Kit (BioVision; Abcam, Waltham, MA, USA) in cell lysates, according to the manufacturer’s instruction. Briefly, cells were resuspended in Cell Lysis Buffer, incubated 10 min at 4 °C, centrifuged and cytosolic extract was used for protein quantification. Next, samples were incubated for 2 h with DEVD-pNA substrate. Caspase-3 activity was assessed by colorimetric detection at 405 nm absorbance using the LT-4000 microplate reader (Euroclone).

### 4.5. Combination Studies 

Drug synergistic effect was performed according to the Chou-Talalay method of synergy quantitation using CompuSyn software [29]. Using a cell survival assay and computerized software data, CI values were generated. A CI of 1 indicates an additive effect between two drugs; a CI greater than 1 indicates antagonism; and a CI less than 1 indicates a synergistic effect (strong: 0.1–0.3; synergism: 0.3–0.7; moderate: 0.7–0.85).

### 4.6. In Vivo Tumor Growth

All mice were bred at the Animal Facility of the Molecular Biotechnology Center (Turin, Italy) and housed under 12 h light/dark cycle, with food and drinking provided ad libitum. Six/eight-weeks-old NOD/SCID/gamma chain^-/-^ (NSG) male mice were s.c. injected in the right flank with 1 × 10^6^ MSTO-211H cells resuspended in 100 μL PBS 1X/matrigel (Matrigel^®^, Corning) solution (ratio 1:1). Tumor growth was measured daily by caliper, according to the equation (L × W^2^)/2, where L = tumor length and W = tumor width. When tumors became palpable, (volume > 40 mm^3^), animals were randomized into four groups (*n* = 9 mice/group) and treated for 28 consecutive days as follows: (1) control group, mice treated with 0.1% DMSO and 10% aqueous propylene glycol solution (vehicle solution for MIA) s.c. and/or physiological solution i.p. (vehicle solution for cisplatin and pemetrexed); (2) MIA-690 (MIA) group, mice treated with 5 μg MIA s.c. daily; (3) cisplatin and pemetrexed (C/P) group, mice treated with 5 mg/kg cisplatin i.p. at days 9 and 23, and 100 mg/kg pemetrexed i.p. at days 3, 5, 9, 18, 23 and 25; (4) MIA-690, cisplatin and pemetrexed (MIA/C/P) group, treated with all three drugs as above. Tumor volumes were monitored, and animals were euthanized 28 days after randomization. Tumors were resected, weighed, and divided for RNA and protein isolation or fixed in 10% buffered formalin. All animal procedures were performed according to institutional guidelines in compliance with national (D.L. N.26, 4 March 2014) and international law and policies (new directive 2010/63/EU). The study was conducted according to the guidelines of the Declaration of Helsinki, and approved by the Italian Ministry of Health (protocol #136/2020-PR 26-01-2020) and by the University of Turin Ethical Committee.

### 4.7. Histochemistry

Sections (3 μm-thick) were cut from formalin-fixed paraffin-embedded (FFPE) tumor tissue blocks and stained with H&E or processed for immunohistochemistry (IHC) analysis. The latter was performed using an automated platform (Ventana BenchMark AutoStainer, Ventana Medical Systems, Tucson, AZ, USA) with the following primary antibodies: anti-Ki-67 (30-9) rabbit monoclonal primary antibody (cat:790-4286, Ventana Medical Systems); cleaved Caspase-3 (Asp175) polyclonal rabbit antibody (cat:9661, Cell Signaling, Danvers, MA, USA). Antigen retrieval was performed using ULTRA Cell Conditioning Solution (ULTRA CC1) antigen retrieval buffer (pH 8.5, Ventana Medical Systems) for all sections and the Ultraview detection system (Roche Diagnostic, Milano, Italy) was used for all analysis. Appropriate positive and negative controls were included for each immunohistochemical analysis. Image acquisition was performed with Hamamatsu NanoZoomer S210 Digital Slide Scanner (Iwata-City, Japan). The number of mitotic cells was assessed by counting number of mitoses per 10 high power fields (mitosis/10HPF) on standard Hematoxylin and Eosin (H&E) stained slides (40 × magnification). Only viable cells were considered in each HPF examined within the section.

### 4.8. Western Blot Analysis

Protein extraction and Western blot analysis were performed as described previously [29,44]. Proteins (30 μg) were separated by SDS-PAGE (10% for P-STAT3, P-NF-κB, p53, P-ERK1/2, and 12% for p21 and BAX), transferred to a nitrocellulose membrane; after blocking with 1% BSA in Tris-buffered saline with 0.1% Tween for 2 h at room temperature, membranes were incubated overnight at 4 °C with the specific antibody (dilution 1:1000 for P-STAT3, P-NF-κB, P-ERK1/2, BAX and 1:500 for p53 and p21). Blots were reprobed with the respective total antibodies or actin (dilution 1:1000 for NF-κB and ERK1/2 and 1:500 for STAT3 and actin) for protein normalization. Immunoreactive proteins were visualized using horseradish peroxidase-conjugated goat anti-mouse, mouse anti-goat (1:4000) or goat anti-rabbit (1:10,000) secondary antibodies by enhanced chemiluminescence substrate (ECL, Bio-Rad, Milano, Italy) using ChemiDoc XRS (Bio-Rad, Milano, Italy); densitometric analysis was performed with Quantity One software (Bio-Rad, Milan, Italy).

### 4.9. Real-Time PCR

Total RNA extraction and reverse transcription to cDNA (1 μg RNA) from PM tumors were performed as described previously [29]. cDNAs were treated with DNA-free DNase (Life Technologies; Thermo Fisher Scientific, Milan, Italy) and reactions performed with 50 ng cDNA, 100 nM of each primer and the Luna Universal qPCR master mix (New England BioLabs, Ipswich, MA, USA) using the ABI-Prism 7300 (Applied Biosystems; Thermo Fisher Scientific). The following primer pairs were used: c-Myc, forward 5′- AGCGACTCTGAGGAGGAACA-3′, reverse 5′-CTCTGACCTTTTGCCAGGAG-3′ (NM_002467.6); COX2, forward 5′-CGGTGAAACTCTGGCTAGACAG-3′, reverse 5′-GCAAACCGTAGATGCTCAGGGA-3′ (NM_000963.4); CyB1, forward 5′-CGAAGATCAACATGGCAGG-3′, reverse 5′-CTTGGAGAGGCAGTATCAACC-3′ (NM_001354845.2); CyD1, forward 5′-ATGTGTGCAGAAGGAGGTCC-3′, reverse 5′-CCTTCATCTTAGAGGCCACG-3′ (NM_053056.3); HMBG1, forward 5′-TATGGCAAAAGCGGACAAGG-3′, reverse 5′-CTTCGCAACATCACCAATGGA-3′ (NM_001363661.2); iNOS, forward 5′-AGACTGGATTTGGCTGGTCCCTCC-3′, reverse 5′-AGAACTGAGGGTACATGCTGGAGCC-3′ (NM_000625.4); MMP2, forward 5′-ACCTGGATGCCGTCGTGGAC-3′, reverse 5′-TGTGGCAGCACCAGGGCAGC-3′ (NM_001302510.1); MMP9, forward 5′-TTGACAGCGACAAGAAGTG-3′, reverse 5′-GCCATTCACGTCGTCCTTAT-3′ (NM_004994.2); VEGF, forward 5′-ATCTTCAAGCCATCCTGTGTGC-3′, reverse 5′-CAAGGCCCACAGGGATTTTC-3′ (NM_001287044.1); 18S rRNA, forward 5′-CCCATTCGAACGTCTGCCCTATC-3′, reverse 5′-TGCTGCCTTCCTTGGATGTGGTA-3′ (NR_146144.1) (designed with the Primer 3 Software, http://www.primer3.org/). The endogenous control used was 18S rRNA. Relative quantification was performed using the comparative Ct (2−ΔΔCt) method.

### 4.10. IGF-1 Analysis 

Tumor xenograft samples were homogenized in RIPA buffer (MilliporeSigma), sonicated and centrifuged at 14,000 rpm (4 °C for 15 min). Total protein lysates were quantified with Bicinchoninic Acid kit (BCA) from (MilliporeSigma). IGF-1 levels were measured following the manufacturer’s protocol using mouse IGF-I ELISA Kit (Abcam, Cambridge, UK), as previously described [29].

### 4.11. Statistical Analysis 

Results are presented as mean ± SEM. Significance was calculated by one-way, or two-way ANOVA followed by Dunnet’s or Tukey’s multiple comparison test for post hoc analysis. Analysis was performed using GraphPad Prism 8.0 (San Diego, CA, USA).

## Supplementary Materials

The following supporting information can be downloaded at: https://www.mdpi.com/article/10.3390/ijms231911248/s1.
