# Supplementary material for: Antagonist of Growth Hormone-Releasing Hormone Potentiates the Antitumor Effect of Pemetrexed and Cisplatin in Pleural Mesothelioma
Source: Int J Mol Sci. 2022 Sep 24;23(19):11248. doi: 10.3390/ijms231911248 (PMC9569772; doi:10.3390/ijms231911248)
Supplement: Supplementary file 1 [file ijms-23-11248-s001.zip › ijms-1889763-supplementary.pdf]

Antagonist of growth hormone-releasing hormone potentiates the antitumor effect of pemetrexed and cisplatin in pleural mesothelioma

Gesmundo et al.

Supplemental Material

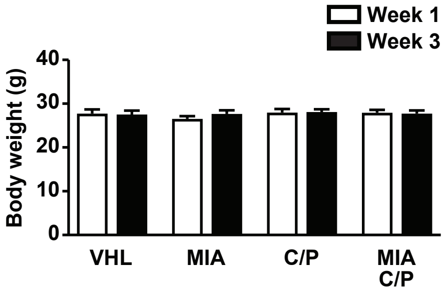

Figure S1. Body weight variation in mice xenografted with MSTO-211H PM cells. Body weight was assessed at week 1 and week 3 in mice treated with vehicle (VHL), MIA-690, cisplatin and pemetrexed (C/P) or the combination of MIA/C/P. Results are mean ± SEM. (n = 9 in each group).
